# AI, universal basic income, and power: symbolic violence in the tech elite's narrative

**DOI:** 10.3389/frai.2025.1488457

**Published:** 2025-02-24

**Authors:** Jean-Christophe Bélisle-Pipon

**Affiliations:** Simon Fraser University, Burnaby, BC, Canada

**Keywords:** universal basic income (UBI), artificial intelligence, Bourdieu, symbolic violence, computational justice, AI ethics, AI governance, AI risk management

## Introduction

In recent years, the concept of universal basic income (UBI) has gained significant attention, not from grassroots community organizations traditionally associated with social welfare advocacy (Jarow, 2023), but from some of the most powerful figures in the technology sector—AI elites (Shead, 2021). Prominent advocates like Elon Musk and Sam Altman argue that UBI is necessary to address the economic disruptions caused by artificial intelligence (AI) and automation (Crumley, 2024). They present UBI as a way to ensure that the benefits of AI are distributed across society, not just concentrated in the hands of a few. However, this seemingly benevolent narrative camouflages a deeper agenda: to seek out a social license to gain public acceptance for the omnipresence of AI in society, and the will to control under the guise of universal benefit.

While economic, social, and normative analyses have been put forward in articles *in Frontiers in Artificial Intelligence* (Ernst, 2022; Huo et al., 2024; Merola, 2022), two key dimensions that remain underexplored in the UBI discussion are 1) the utilitarian calculation behind the AI-justified UBI narrative; and 2) the associated symbolic violence, as articulated by sociologist Pierre Bourdieu. I argue that UBI, while ostensibly a tool for social good, may end up justifying even greater disparities in wealth and entrench symbolic violence by reinforcing divisions between AI owners, those skilled or capacitated in using AI, and those who are merely recipients of its “benefits.” This symbolic violence is particularly perverse as it perpetuates a narrative of AI as universally beneficial, when in reality, it risks exacerbating socio-economic inequalities and creating profound epistemic and symbolic injustices.

## The AI elites' advocacy for UBI

The advocacy for UBI by AI elites is a relatively new phenomenon. Figures like Elon Musk, the CEO of Tesla, SpaceX, and X, and Sam Altman, the CEO of OpenAI, have positioned themselves as champions of UBI. Musk (2024) recently indicated about the rise of AI that “In a benign scenario, probably none of us will have a job. There would be universal high income. There would be no shortage of goods and services. The question will really be one of meaning: if a computer can do, and the robots can do, everything better than you, does your life have meaning? I do think there's perhaps still a role for humans in that we may give AI meaning.” For his part, Altman (2016) indicated that “[he's] fairly confident that at some point in the future, as technology continues to eliminate traditional jobs and massive new wealth gets created, we're going to see some version of this at a national scale.” AI elites argue that as AI and automation increasingly replace human labor, UBI will be essential to prevent widespread economic dislocation and social unrest. This argument may be compelling, especially in a world where technological advancements threaten to render large segments of the workforce obsolete (Islam, 2024). However, the promotion of UBI by these tech magnates is not simply a philanthropic gesture; it is deeply intertwined with their interests in the expansion and dominance of AI technologies. Crane et al. (2019) argue that corporate strategies often align with maintaining and enhancing power structures that benefit corporate elites. The advocacy for UBI by AI leaders can be seen as a strategic move to pre-emptively address potential backlash against AI-induced risk and negative externalities, such as job losses or job polarization [i.e., reducing middle wages, shifting demand toward low and high wages (see Goos and Savona, 2024)], thereby securing a favorable business environment for continued AI development and deployment.

Without going so far as to say that AI may be an existential risk (or X-Risk, a risk to the very viability of humanity)—as other movements such as the members of the effective altruism movement and the associated cause of longtermism may do (Jecker et al., 2024)—AI may pose significant economic and social risks if job losses are not offset. The narrative presented by these AI leaders suggests that UBI is a necessary adaptation to the inevitable rise of AI—a tool to ensure that everyone benefits from technological progress. Yet, this narrative serves to legitimize and reinforce the power dynamics that already exist in the AI industry. By advocating for UBI, these AI elites position themselves as benevolent visionaries who are concerned about the wellbeing of humanity. However, as Sadowski (2016) argues, promoting UBI can be a strategic way for AI elites to deflect criticism, maintaining control over narratives about AI's future while avoiding challenges to their profit motives. This framing distracts from the fact that the same individuals who are pushing for UBI are also those who stand to gain the most from the proliferation of AI technologies (Spencer, 2024). Bourdieu's concept of symbolic violence offers valuable insight into the deeper implications of UBI in the context of AI; however, it is important first to examine UBI from its utilitarian foundation.

## A utilitarian justification for UBI

This narrative aligns with a utilitarian view for assessing the benefits and risks of AI in society. AI elites apply a utilitarian calculation, evaluating the moral justification of replacing humans with AIs by weighing the potential to maximize societal wellbeing against the associated harms. From this eudemonic standpoint—focused on balancing wellbeing and harm, or even more simply pleasures and pains—they envision a future where AI's dominance across human-dominated fields leads to a society characterized by widespread leisure and, for some, heightened performance. In their view, this transformation is morally defensible if measures (such as UBI) are implemented to mitigate the negative effects and ensure distribution of certain benefits for all. UBI is thus used to justify the possibility, and to demonstrate, that AI can provide for humanity's basic needs, while at the same time justifying that some can be ultra-wealthy and possess these technological tools of humanity's (apparent) sustenance (Islam, 2024). While it has not been directly invoked up to now, this is a curious application of John Rawls' principle of difference, which in his “Theory of Justice” states that social and economic inequalities are to be arranged so that they are to the greatest benefit of the least advantaged members of society, consistent with the just savings principle and the principle of fair equality of opportunity (Rawls, 1971). Arguably, providing UBI to all does not solve everything; it creates more equality amongst the less well-off, without acting to address inequalities and wealth gaps. Yes, this would be a first for humanity—an economic safe net from which all could benefit (which appears to be of a fixed-benefit nature, with no indication of adjustment to economic trends)—but this cannot justify the kind of leanness in which it seems to place the non-owners of AI (i.e., virtually the entire world population) compared to the AI elites. It is hard to make a convincing utilitarian claim that this is for the benefit of the less well-off. Furthermore, as Sen (2009) argues, a focus on utility maximization may neglect the distribution of capabilities and freedoms, which are essential for genuine social justice; something very plausible if like Musk (2024) is thinking that if “[computers and robots can be doing] everything better than [humans], does [human] life have meaning?” With AI potentially representing a X-risk (Jecker et al., 2024) or at the very least risking to lead to a “a shift in power toward actors with the capital and authority to deploy powerful AI systems, such as elites, corporations, and governments” (Dafoe, 2018), it is very unclear that AI will actually maximize utility and be for everyone's benefits.

Unfortunately for AI-justified UBI proponents, a study funded by Altman has found that UBI is not a comprehensive solution to the economic challenges posed by AI-driven job loss (Ropek, 2024). The research, conducted by OpenResearch (2024) between 2020 and 2023, provided $1,000 a month to 1,000 low-income individuals, with a control group receiving $50 monthly. While UBI helped participants cover essential expenses like housing and groceries, it did not lead to significant improvements in employment quality, education, or overall health. The study concluded that while UBI can alleviate some immediate financial stress, it falls short of addressing deeper systemic issues such as healthcare access, job stability, and upward mobility. Thus, UBI alone is unlikely to mitigate the broader economic impacts of AI on the workforce. So, unfortunately, proponents' utilitarian calculation does not seem to be working as well as they would like. As a result, the impetus for supporting UBI seems more ideological and self-serving than beneficial.

## Symbolic violence in the AI-justified UBI narrative

Here, Pierre Bourdieu's concept of “violence symbolique” can help deceive AI elites' benevolent narrative. *Symbolic violence* refers to a form of domination that is subtle and often imperceptible, yet profoundly effective in maintaining social hierarchies (Bourdieu and Wacquant, 1992). Symbolic violence operates through the imposition of meanings that are accepted as legitimate, even by those who are subordinated by them (Bourdieu, 1993). This form of violence is not physical, but it is deeply embedded in the social structures and cultural norms that shape our understanding of the world. With society's increased digitalization, Couldry and Mejias (2020) denote how data practices can constitute a new form of colonialism, reinforcing existing power structures through symbolic means. Such symbolic violence allows dominant groups perpetuate their power without overt coercion, by making their worldview appear natural and inevitable.

In the context of UBI and AI, symbolic violence manifests in the way that the narrative of AI, as a universal good, is constructed and disseminated. The AI elites' promotion of UBI suggests that the best way to address the disruptions caused by AI is to provide people with a guaranteed basic income, thereby ensuring that everyone benefits from technological progress. However, this narrative obscures the deeper structural inequalities that are being reinforced by the same technologies that UBI is supposed to mitigate. Symbolic violence, Bourdieu and Wacquant (1992, p. 172) notes, “accomplishes itself through an act of cognition and of misrecognition that lies beyond—or beneath—the controls of consciousness and will.” In the case of AI-justified UBI, public's acceptance of this proposal as a universal good would be a form of misrecognition that a symbolic violence is being perpetrated and instead considering that AI and UBI are normal and rather logical within the existing social order. Such acceptance would be a legitimization of the power of the AI elite by presenting UBI as the solution to the very problems their technologies create, thus reinforcing the existing social order.

## AI-justified UBI as a tool for maintaining social hierarchies

UBI, as promoted by AI elites, can be seen as a tool of symbolic violence in several ways. First, it reinforces the division between those who own and control AI technologies and those who are merely consumers of its benefits. The owners of AI—who are also the primary advocates of UBI—are positioned as the benevolent providers of a safety net for the masses. Meanwhile, the recipients of UBI are cast as passive beneficiaries of a system that they have little control over. This dynamic perpetuates the power of the AI elite, while simultaneously legitimizing their dominance by presenting them as the solution to the very problems that their technologies create.

Moreover, UBI as a form of symbolic violence operates by masking the true nature of the inequalities that it purportedly seeks to address. By providing a basic income, the narrative suggests that the economic and social disruptions caused by AI can be managed and mitigated. However, this narrative ignores the fact that UBI does nothing to address the underlying power imbalances that give rise to these disruptions in the first place. Critics argue that UBI, without accompanying structural reforms, may fail to address underlying inequalities (Parijs and Vanderborght, 2017), just like the OpenResearch study hinted. As Jarow (2024) puts it, “hitching the case for basic income to fears of rapid AI progress makes it far more vulnerable than it needs to be.” By linking UBI to AI, its advocates risk creating a policy that merely manages the symptoms of economic inequality without addressing the root causes. This approach perpetuates a superficial solution that maintains the status quo, allowing the AI elite to continue accumulating wealth and power while the majority remains dependent on the systems that marginalize them.

## The perverse nature of symbolic violence in UBI

The symbolic violence inherent in the promotion of UBI by AI elites is particularly perverse because it creates the illusion of inclusivity and fairness; values central to AI ethics (Victor et al., 2024). The narrative of UBI as a universal good suggests that everyone stands to gain from the increased presence of AI in our societies. However, this narrative obscures the fact that the benefits of AI are not distributed equally, and that UBI, as currently envisioned, may actually entrench existing inequalities rather than alleviate them. By framing UBI as a necessary response to AI-induced unemployment, the AI elites are effectively shifting the focus away from the need for more equitable distribution of power and resources. Those who control AI technologies continue to benefit disproportionately, while those who are dispossessed by these technologies are offered only a minimal safety net in return.

Moreover, this symbolic violence has epistemic implications (Bourdieu, 1993), as it shapes our understanding of what is possible and desirable in a world increasingly dominated by AI. Musk (2016), almost a decade ago, said that “There's a pretty good chance we end up with a universal basic income, or something like that, due to automation. I'm not sure what else one would do. I think that is what would happen. People will have time to do other things, more complex things, more interesting things. Certainly more leisure time.” The promotion of UBI by AI elites reinforces the idea that the best we can hope for is an AI-induced universal income, rather than a more radical rethinking of how wealth and power are distributed in society—nor seeing UBI as a way to enhancing people's capabilities (Endo and Choi, 2024). The loss of meaningful work can have profound psychological effects, as Jahoda (1982) highlights the role of employment in providing structure, social contacts, and a sense of purpose. Rubin (2024) presents it nicely in indicating that “The AI revolution is accentuating the flow of income and power to the owners of property, leaving a new class—the precariat—wallowing in insecurity and existential fear.” Or as Jarow (2024) puts it “The basic income movement might be better off severing ties with speculations about AI altogether. Then, the conversation could focus on what basic income can actually be: an effective anti-poverty tool that would neither stave off dystopia nor usher in a leisurely paradise, but instead, just a world with less poverty.“ AI-justified UBI's narrative acts as symbolic violence and limits our collective imagination, making it harder to envision alternative futures where technology actually serves the common good rather than aiming to place populations in a state of indigence compared to the fortunes of those who control AI.

## Discussion

Universal Basic Income, as promoted by AI elites, is not the straightforward solution to the disruptions caused by AI that it is often portrayed to be. Instead, it can be understood as a form of symbolic violence that reinforces existing power dynamics and socio-economic inequalities. Or as Bourdieu would put it, those in power “tend to seek social respectability” (Bourdieu and Passeron, 1970) and this can be achieved through the imposition of narratives and meanings presented as legitimate while concealing the power relations which are the basis of their force (Bourdieu, 1987). By presenting UBI as a benevolent response to AI-induced unemployment, the AI elite mask their own role in creating the very problems that UBI is supposed to solve. In doing so, they perpetuate a narrative that benefits them while marginalizing those who are most negatively affected by the rise of AI. Interestingly, what is being distributed is a basic economic safety net, without committing to providing basic and free access to AI itself. Recent statements by OpenAI suggested that free AI models may not be here to stay. The current idea is a freemium model with advertising to better monetize the models, development costs, and hosting (FT News Briefing, 2024). Dayan et al. (2024) demonstrated that older AI models tend to experience performance degradation over time, which, although the term “dementia” anthropomorphizes AI, effectively illustrates the decline of these models. Therefore, offering (un)restricted access to older AI models is not a viable solution to share more broadly the benefits of AI or promote computational justice. This is especially true since making such models widely available may not ensure equitable access, particularly when those who can afford newer models receive them significantly earlier than others.

However, what is touted as benefits falls short of addressing structural inequities or advancing computational justice, which goes beyond mere access to AI. Computational justice emphasizes equitable access, representation, and outcomes in AI, ensuring that everyone—regardless of socioeconomic status or geography—can not only use AI but also participate in its development and governance. It requires addressing biases in algorithms, democratizing computational power (so actively supporting computational justice), and ensuring transparent, ethical governance. Such an approach could empower marginalized communities and provide tools to tackle systemic inequities. Instead, these economic models appear more focused on sustaining AI's pervasive presence in everyday life, potentially prioritizing corporate profit over the transformative potential of AI to create a more equitable digital society both economically for all and in making the AI tools accessible for all. By sidelining these principles, the freemium model risks cementing existing inequalities rather than challenging them, raising concerns about whether the AI-driven future will be one of inclusion or exploitation. this will lead to a state where people will receive a UBI as a justification for the increased presence of AIs in society and to compensate for the externalities this induces, then will either have to pay for the advanced models or watch advertising to access the basic models. To truly address the societal disruptions posed by AI, structural reforms are necessary, including policies that promote equitable distribution of wealth and power (Stiglitz, 2019).

This framing of UBI as a panacea for AI-induced challenges reflects a broader strategy by AI elites to deflect criticism and maintain control over narratives about AI's future. As Schiff (2023) argues, the ethical aspirations embedded in AI policy often fall short in their translation to actionable solutions due to challenges like technical feasibility, value acceptability, and institutional constraints. By focusing on symbolic solutions like UBI, which align with their profit motives, AI elites can sidestep calls for deeper structural reforms that would redistribute wealth or power. Ethical principles, while rhetorically emphasized, are often narrowed or deprioritized when translated into sector-specific policies, reflecting institutional limitations and a preference for technical fixes over transformative socio-political solutions. This narrowing of ethical commitments not only limits meaningful progress but also exacerbates the phenomenon of AI ethics dumping, where ethical responsibilities are shifted from developers and regulators onto ill-equipped users and local communities (Bélisle-Pipon and Victor, 2024). AI-driven UBI serves as a key example of how symbolic solutions allow AI elites to divert attention from their complicity in creating structural inequalities. It enables them to project a narrative of benevolence while avoiding substantive changes that would challenge their profit motives or operational frameworks. Ethics dumping is particularly insidious in this context because it disguises systemic inequities under ethical innovation. Developers embed normative assumptions into AI systems, and high-level ethical guidelines fail to account for local contexts, leaving the most vulnerable communities to grapple with the downstream impacts of these technologies. The promotion of UBI thus reinforces a cycle where the burdens of AI-induced disruptions are offloaded onto those least equipped to address them, all while AI elites continue to benefit from an unchallenged status quo.

Furthermore, UBI as a mitigating mechanism remains predominantly confined to the United States—or more specifically, to key regions where AI development and control are concentrated among AI elites—while failing to extend its scope to the global population. This narrow framing is highly problematic given that AI systems are trained on data sourced from diverse global populations and have profound, far-reaching effects not only on humanity as a whole but also on the environment (Crawford, 2021). Framing the benefits of AI within the privileged contexts of already-advantaged nations disregards the inequitable realities of the Global North and Global South divide (Birhane, 2021; Mohamed et al., 2020), particularly from the perspective of an AI-driven UBI. Such an exclusionary focus not only marginalizes billions of individuals who contribute to the ecosystems enabling AI but also underscores a critical flaw in the utilitarian logic underpinning the justification for AI development. The omission of global equity considerations exposes the ethical limitations of benefit-sharing mechanisms like UBI, raising serious questions about the moral defensibility of AI's promised benefits and the structural inequities they perpetuate.

Tackling the issues surrounding AI governance requires embedding ethical principles into practical frameworks that consider the broader socio-political context of technological innovation (Bélisle-Pipon et al., 2022). Policymakers must move beyond symbolic gestures, such as Universal Basic Income (UBI), and instead focus on participatory decision-making, context-specific solutions, and robust accountability mechanisms. These measures must seek to dismantle the structural inequalities perpetuated by prevailing AI narratives, ensuring that technological progress is guided by principles of justice and equity rather than being wielded as a tool for consolidating power and profit. The expanding influence of tech elites in governmental and political spheres, exemplified by figures like Elon Musk's involvement in federal policymaking and Sam Altman's forays into municipal politics, highlights a calculated effort to control AI's development and its societal implications. This trend aligns with concerns over an impending “AI regulation winter” (Bélisle-Pipon, 2024) scenario in which regulatory mechanisms are intentionally weakened to serve elite interests, further entrenching their dominance. This pervasive involvement across federal, municipal, and regulatory levels underscores a systematic strategy to shape society's future in ways that prioritize private power over public good.

In this context, UBI risks becoming a superficial concession—a mechanism for placating the public while masking deeper systemic inequities. As such, it can function as a form of symbolic violence that reinforces structural injustices rather than addressing them. Framed merely as a token redistribution of wealth, UBI has the potential to serve as a veneer of reform, obscuring the underlying exploitation and inequity facilitated by unchecked AI expansion and aggravated computational injustices. Policymakers must reject deregulation or the outsourcing of AI governance to elites whose primary aim is to entrench their dominance. Instead, they should adopt comprehensive strategies that aim at providing social conditions that enable individuals and collectives to thrive (Lees-Marshment et al., 2020), which include progressive taxation to redistribute AI-generated wealth, substantial investment in education and workforce reskilling to equip individuals for an AI-driven economy, stringent labor regulations to protect workers from exploitation in automated industries, and ensure that AI is a commons rather than adding to socio-economic inequalities, a growing computational injustice. Critically, there must be a concerted effort to challenge the disproportionate influence of tech elites on public policy (Ricaurte, 2022), ensuring that governance frameworks are designed to serve collective wellbeing rather than elite agendas. Superficial solutions like AI-funded UBI must not be heralded as quick fixes for systemic inequalities. Instead, efforts must focus on addressing the root causes of these disparities, building a society where technological innovation supports justice, inclusion, and equity rather than perpetuating existing power imbalances.
